# The long-term avoided recurrences and recurrence-related cost of alectinib for postoperative adjuvant therapy in Chinese patients with early-stage ALK-positive non-small cell lung cancer

**DOI:** 10.3389/fpubh.2025.1571980

**Published:** 2026-02-05

**Authors:** Xuanxuan Yan, Sijuan Zhou, Jiahao Hu, Yan Xia, Haotian He, Nick Jovanoski, Melina Arnold, Jian Hu, Pingping Song, Benyuan Jiang, Yong Zhang, Jingwen Lu, Yilong Wu, Xiaohua Ying

**Affiliations:** 1 Department of Health Economics, School of Public Health, Fudan University, Shanghai, China; 2 Shuguang Hospital Affiliated With Shanghai University of Traditional Chinese Medicine, Shanghai, China; 3 Shanghai Health Development Research Center (Shanghai Medical Information Center), Shanghai, China; 4 Shanghai Roche Pharmaceuticals Ltd., Shanghai, China; 5 F. Hoffmann-La Roche Ltd., Basel, Switzerland; 6 Department of Thoracic Surgery, The First Affiliated Hospital of Medical School of Zhejiang University, Hangzhou, China; 7 Shandong Cancer Hospital and Institute, Shandong First Medical University and Shandong Academy of Medical Sciences, Jinan, China; 8 Guangdong Provincial People's Hospital, Guangdong Academy of Medical Sciences, Guangdong Lung Cancer Institute, Guangzhou, China; 9 Department of Pulmonary and Critical Care Medicine, Zhongshan Hospital, Fudan University, Shanghai, China; 10 Faculty of Physical Education, Fudan University, Shanghai, China; 11 NHC Key Laboratory of Health Technology Assessment, Shanghai, China

**Keywords:** alectinib, ALK-TKI, NSCLC, China, Markov, drug outcomes

## Abstract

**Aims:**

Alectinib was approved by the US, Europe and China in 2024 as the first adjuvant targeted therapy for ALK+ NSCLC, lowering risk of disease recurrence or death by 76%. Alectinib addresses a critical gap in postoperative adjuvant therapy for ALK+ NSCLC. From Chinese healthcare-system perspective, this study evaluates the impact of introducing alectinib in adjuvant therapy for stage IB (tumor ≥ 4 cm) to IIIA (UICC/AJCC 7th edition) ALK+ NSCLC on prevention of recurrence and the associated direct medical costs, compared to platinum-based chemotherapy.

**Methods:**

A Markov model was developed to estimate the number of locoregional and metastatic recurrences over a 10-year period by defining four health states: disease-free survival, locoregional recurrence, metastatic recurrence, and death. In the control group, all patients received platinum-based chemotherapy, while in the intervention group, 75% received alectinib and 25% received platinum-based chemotherapy. Clinical data were collected from open-label, randomized phase 3 trials ALINA and ALEX. Cost parameters were derived from local charges, expert consultation, and published literature.

**Results:**

Compared to control group, the intervention group would reduce recurrences by 11,300 cases over 10 years, including 3,684 locoregional and 7,616 metastatic cases. This corresponds to a 45.82% lower recurrence rate. Estimated recurrence-related cost savings amounted to 6.910 billion RMB, with 1.445 billion RMB saved from locoregional recurrences and 5.465 billion RMB from metastatic recurrences. This represents a 41.49% reduction in costs compared to control group. These findings were robust across various scenario analyses.

**Conclusion:**

Using alectinib in postoperative adjuvant therapy significantly reduces both the recurrence rate and recurrence-related treatment costs for stage IB (tumor ≥ 4 cm) to IIIA ALK+ NSCLC patients, compared to platinum-based chemotherapy. From perspective of Chinese healthcare system, this approach shows substantial potential for preventing recurrence and achieving cost savings.

## 1 Introduction

Lung cancer is the leading cause of cancer-related deaths worldwide, with non-small cell lung cancer (NSCLC) comprising for approximately 85% of all cases. In China, the incidence of lung cancer continues to rise, with an estimated 1.06 million new cases annually, making it a critical public health concern. Early screening has led to an increasing proportion of early-stage NSCLC cases, which now exceeds 50% (1, 2). Despite early diagnosis, these patients are faced with a substantial risk of postoperative recurrence, with average recurrence-free survival of 18.8 months and 5-year survival rate of only about 50% (3, 4). Current standard of care for early-stage resectable NSCLC patients is adjuvant platinum-based chemotherapy, which yields the 5-year overall survival (OS) by approximately 5.4% (5). Among the subtypes of NSCLC, ALK-positive NSCLC (ALK+ NSCLC) is defined by the presence of a fusion gene involving the anaplastic lymphoma kinase (ALK), occurring in 2 to 11% of NSCLC cases (6–10), particularly among younger patients with minimal or no smoking history. Compared to other subtypes, ALK+ NSCLC has characteristics such as early age of onset, a high risk of brain metastasis, elevated recurrence risk, and poor prognosis (11). Recently, the ALINA phase III clinical trial demonstrated the clinical efficacy of alectinib as an adjuvant therapy for early-stage, resectable ALK+ NSCLC patients. In April 2024, alectinib was globally approved for the first time as an adjuvant targeted therapy after surgery in ALK+ NSCLC. This approval, which reduces the risk of tumor recurrence or death by 76%, marked a major advancement in filling the gap in adjuvant treatment options for the patients. On June 28, 2024, the China National Medical Products Administration officially approved adjuvant indication for stage IB (tumor ≥4 cm) to IIIA (UICC/AJCC 7th edition, similarly hereinafter), further validating the clinical benefits of alectinib.

While alectinib has been extensively studied and proven effective in advanced ALK+ NSCLC (12–15), evidence regarding its economic impact, particularly for early-stage patients, remains limited. This study aims to construct a treatment impact model for adjuvant therapy in stage IB (≥4 cm) to stage IIIA ALK+ NSCLC patients from the healthcare system perspective of, assessing the reduction in recurrence and direct medical costs by using alectinib for adjuvant therapy after surgery compared to chemotherapy alone. The findings are expected to provide evidence on the long-term benefits of alectinib as adjuvant therapy for early-stage resectable ALK+ NSCLC patients, highlighting its role in reducing recurrence risk, and alleviating the burden on patients and society.

## 2 Materials and methods

### 2.1 Target population

This study identified the target patient population as stage IB (tumor ≥4 cm) to stage IIIA ALK-positive (ALK+) NSCLC patients with resectable tumor and undergoing adjuvant therapy. Relevant epidemiological parameters were collected to support this estimation.

According to the annual report of the China Cancer Center, there were 1.06 million new lung cancer cases in 2022 (16), with approximately 85% classified as NSCLC (17). The annual growth rate of lung cancer incidence in China was 0.8% (18). This study used the compound annual growth rate method to predict the number of new NSCLC cases in China from 2025 to 2034. Additionally, based on previous studies of the Chinese population, the ALK+ rate among NSCLC patients was assumed to be 5.14% (10).

The model was based on real-world data from published literature and expert interviews to estimate the proportion of patients diagnosed with stage IB (tumor ≥4 cm) to stage IIIA NSCLC (19–21), the proportion of patients with resectable disease, and those receiving adjuvant therapy post-surgery (see Table 1). Since the abovementioned data did not specify the tumor size among stage IB patients, the model assumed that one-third of stage IB patients have tumors larger than 4 cm. This assumption was validated by multiple clinical experts and was expected to remain constant over time.

**Table 1:** Proportion of operable NSCLC patients receiving adjuvant therapy by disease stage.

Disease stage	Proportion of ALK+ NSCLC patients	Proportion of operable patients	Proportion of patients who receive adjuvant treatment conditional on being operated
IA	12.0%	75.1%	23.7%
IB (tumors <4 cm)	4.2%	75.1%	23.7%
IB (tumors ≥4 cm)	1.8%	75.1%	23.7%
IIA	2.9%	73.2%	45.8%
IIB	2.9%	73.2%	45.8%
IIIA	12.9%	62.6%	64.5%
IIIB/IV	63.5%	28.5%	56.8%

### 2.2 Model overview

Based on similar studies and in conjunction with the “China Guidelines for Pharmacoeconomic Evaluations,” a semi-Markov model was developed from the perspective of the Chinese healthcare system. The model focused on stage IB (tumor ≥4 cm) to stage IIIA ALK+ NSCLC patients receiving adjuvant therapy post-surgery. It estimated the impact of alectinib adjuvant targeted therapy versus platinum-based chemotherapy on recurrence cases and associated treatment costs for resectable patients. Epidemiological, clinical, and cost parameters necessary for model construction were collected.

**Figure 1:**
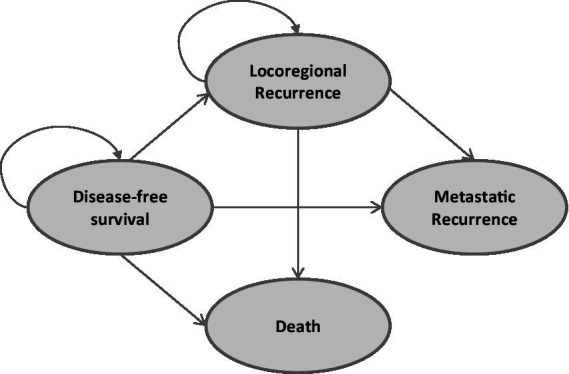
Markov model structure.

**Figure 2:**
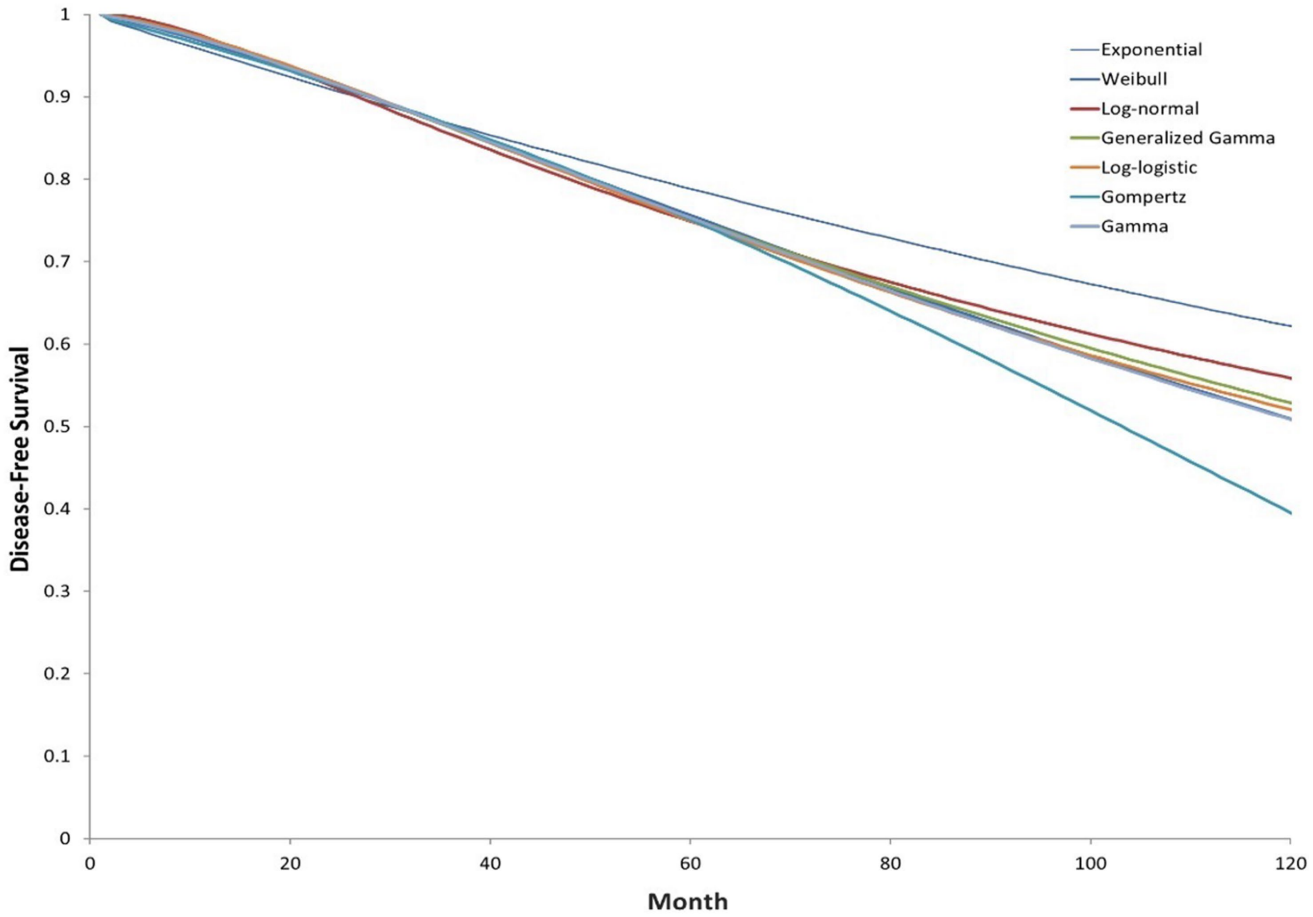
Extrapolated investigator-assessed DFS curves (ALINA; clinical cutoff: June 26, 2023; intervention group).

**Figure 3:**
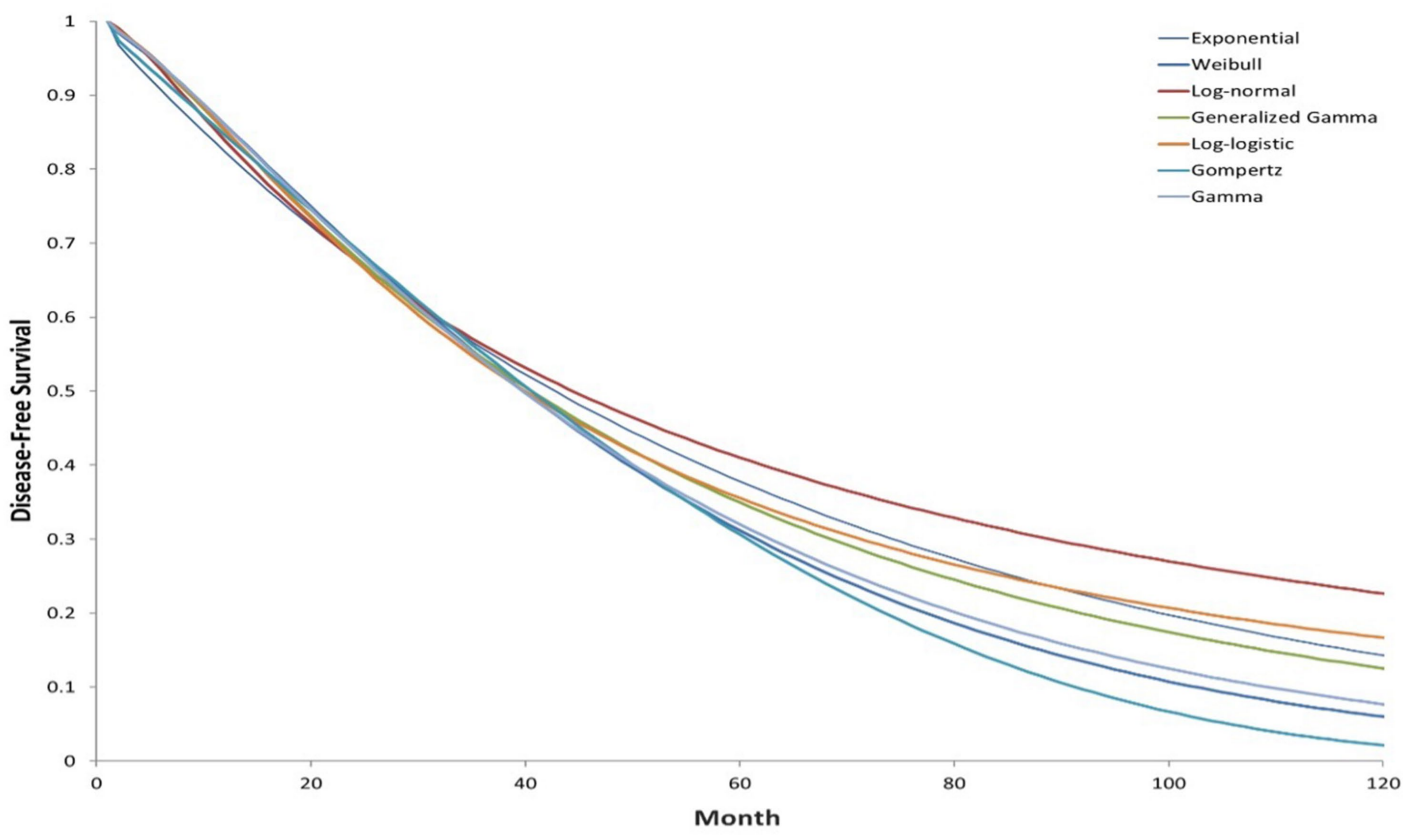
Extrapolated investigator-assessed DFS curves (ALINA; clinical cutoff: June 26, 2023; control group).

A Markov model was employed to simulate patients’ transitions among different health states, including disease-free survival (DFS), locoregional recurrence, metastatic recurrence, and death (Figure 1). In this model structure, time-dependent transition probabilities were derived directly from the parametric survival curves fitted to the clinical trial data (as shown in Figures 2, 3), rather than using fixed probabilities for each cycle. Clinical data were primarily derived from phase III randomized trials ALINA (22) and ALEX (13). Transition probabilities between health states were estimated using data from DFS events reported in the ALINA trial, progression-free survival (PFS) events from the ALEX trial, and the study by Nakamichi et al. (23). Health state transitions were modeled over a 10-year horizon to estimate long-term outcomes, with a 1-year cycle length. In the base case analysis scenario, all patients in the control group received platinum-based chemotherapy, while 75% of patients in the intervention group received alectinib, and 25% received platinum-based chemotherapy.

In the base-case analysis, we assumed that 75% of patients in the intervention arm would receive adjuvant alectinib. This assumption was justified based on three considerations: (i) the current and anticipated penetration of ALK testing in resected early-stage NSCLC patients in China, (ii) the formulary and reimbursement status as well as hospital-level accessibility of alectinib in territorial hospitals, and (iii) observed patterns of clinical adoption following approval of the adjuvant indication. Therefore, the assumed uptake rate was validated through consultation with Chinese clinical experts.

### 2.3 Calculation of patients in different health states

DFS data for patients receiving alectinib adjuvant targeted therapy and adjuvant chemotherapy were obtained from the ALINA phase III clinical trial. Within the model, the probabilities of transitioning from the disease-free state to locoregional recurrence, metastatic recurrence, or death are calculated based on the DFS data from patients in the adjuvant alectinib and chemotherapy arms of the clinical trial. Although a single case of a new primary lung cancer was observed in the ALINA trial, this event was excluded from the model due to the uncertain relationship between the new and original malignancies. The Kaplan–Meier curves for patients in the alectinib adjuvant targeted therapy group and platinum-based chemotherapy group, as assessed by researchers in the ALINA study, are available for approximately 50 months (cutoff date: June 26, 2023), and parametric models were used to extrapolate (see the Supplementary material). Under the proportional hazards (PH) assumption, the analysis aggregated patients in the study groups, using a covariate to capture the group to which each patient belonged, to model the effect of alectinib adjuvant targeted therapy on the location parameter for each group. The log-cumulative hazard plot of DFS assessed by researchers, and the Schoenfeld test supporting this conclusion (see the Supplementary material). The final selection of parameter distribution for long-term fitting was based on the goodness of fit between predicted and observed data, as well as the clinical validity of long-term predictions. Based on the Akaike Information Criterion (AIC) and the Bayesian Information Criterion (BIC) criteria, the log-logistic distribution demonstrated the best fit and was selected for extrapolation (Figures 2, 3).

The median follow-up time in the ALINA study was 32 months. Given that most recurrences occur within 5 years, the long-term DFS prediction may underestimate long-term DFS (24). To address this, patients were assumed to be cured if they remained disease-free for 5 years. A literature review was conducted on the conditional disease-free survival (cDFS) in early-stage NSCLC patients who had undergone resection to gather evidence on the proportion of patients who may continue to experience recurrence or disease-related death after a period of disease-free survival. Studies have shown that among patients who remained disease-free for 3 years, the 5-year disease-free survival rate was 91% for stage I patients and 83% for those with stage II or higher (25). However, this study only focused on Korean patients, and it is unclear what proportion of their sample was ALK+. To validate this evidence, clinical expert consultations were conducted, concluding that patients who were disease-free for 5 years could be considered cured, though five experts believed that 0–10% of these patients might still recur, and two experts believed that 20–30% might still recur. In the base-case analysis, the model assumed that all patients who remain disease-free for 5 years are considered cured.

The model also considered that patients who initially experienced locoregional recurrence may subsequently progress to metastatic recurrence. PFS data were used to capture this aspect. Similar to the DFS, long-term PFS predictions also employed parametric models, with the log-normal model providing the best fit, based on the data form the ALEX phase III clinical trial and Nakamichi et al. (23). Table 2 shows the PFS for patients who received radiotherapy and chemotherapy for locoregional recurrence, with progression-free events leading to disease progression or death sourced from the ALEX (13). Additionally, since there was no evidence indicating differences in treatment or efficacy for locoregional recurrence between patients receiving platinum-based chemotherapy and those receiving alectinib adjuvant targeted therapy, the PFS data were assumed identical for both the intervention and control groups.

**Table 2:** Progression-free survival after non-metastatic recurrence.

Year	Progression-free survival
1	74%
2	44%
3	27%
4	17%
5	11%
6	8%
7	6%
8	4%
9	3%
10	2%
Proportion of patients who experience disease progression versus death *	88.9%

* ALEX (ITT, intervention arm, clinical cutoff: November 30, 2018) (23).

### 2.4 Treatment costs

A literature review of both domestic and international guidelines related to the treatment of non-small cell lung cancer (NSCLC) was conducted, including the “2024 NCCN Clinical Practice Guidelines in Oncology: Non-Small Cell Lung Cancer” and the “2024 CSCO Guidelines for the Diagnosis and Treatment of Non-Small Cell Lung Cancer.” In combination with clinical expert interviews and literature data, the main treatment patterns for ALK+ NSCLC patients were outlined, and the real-world costs of recurrence treatments were calculated. The cost parameters mainly involve the treatment costs for locoregional recurrence and metastatic recurrence, which encompass treatment regimens and their respective proportional, drug and surgical costs, adverse events (AEs) (see the Supplementary Tables 1A, 2A, 3A) and follow-up cost parameters for inpatient and outpatient care. Data sources and assumptions were validated to ensure consistency with clinical guidelines and real-world practices, reflecting the economic impact of recurrence treatments in the target population.

### 2.5 Treatment regimens and proportions

Through expert consultations and reviews of clinical guidelines, the treatment regimens and their proportional usage for locoregional and metastatic recurrence in ALK+ NSCLC patients were determined. These proportions were used to calculate the associated treatment costs, as detailed in Table 3.

**Table 3:** Treatment regimen and proportion of patients with ALK+ NSCLC.

Recurrence type	Main treatment options	Proportion of treatment regimen (%)	Source of treatment proportion
Locoregional recurrence	1. Surgery	3.00%	Expert consultation
	2. Chemoradiotherapy	12%	
	3. ALK inhibitor (alectinib)	45.00%	
	4. Surgery + ALK inhibitor	7.00%	
	5. ALK inhibitor + chemoradiotherapy	33.00%	
Metastatic recurrence	1. Chemotherapy	5.00%	
	2. ALK inhibitor (alectinib)	55.00%	
	3. ALK inhibitor (lorlatinib)	30.00%	
	4. ALK inhibitor (brigatinib)	5.00%	
	5. ALK inhibitor (ensartinib)	5.00%	

For metastatic recurrence, it should be noted that for patients with brain metastases, additional direct medical costs compared to non-brain metastases need to be considered, and specific treatment plans were not included.

If patients experienced recurrence during the initial alectinib adjuvant targeted therapy period, it may be due to the development of resistance or other factors, which would affect the future use of alectinib and necessitate a switch to another ALK inhibitor. Through expert consultations and review of guidelines, it is recommended that 90% of cases switch to Lorlatinib, 5% to Crizotinib, and another 5% to Brigatinib. These recommendations ensured that post-recurrence treatment aligned with current clinical practices and addresses drug resistance effectively.

### 2.6 Specific cost parameter settings

The specific cost parameters for drugs, surgical procedures, AEs management and follow-up care (both inpatient and outpatient) associated with the treatment regimens are detailed in Table 4. It should be noted that 24–30% of ALK+ NSCLC patients present with brain metastases at the time of initial diagnosis (26), with 27% adopted as the base-case assumption. The cost parameters were derived from a combination of expert consultations, published literature, government and hospital price lists, and databases such as Yaozhi website^1^ in China. While the unit price of alectinib is subject to a confidentiality agreement, the cost parameter used in this model is derived from official and verifiable sources within the Chinese healthcare system. To further address price uncertainty and enhance transparency, a scenario analysis of ±20% on this parameter was conducted to assess its impact on the overall results.

**Table 4:** Treatment cost parameters (RMB, ¥).

Drug/surgical cost parameters	Parameter value	Range	Drug dosage	Duration of medication/follow-up frequency	Source
Chemotherapy (pemetrexed + cisplatin/carboplatin)	4,790/person/cycle	(2,500, 12,500)	4 cycles	—	Government/hospital published prices, Yaozhi website (27)
Radiotherapy	600/Gy	(600, 1,000)	Total treatment dose: 50 Gy	—	Expert consultation
Surgery *	46,345/person/year	(40,000, 50,000)	1 surgery	—	Li et al. (28)
Alectinib	Confidentiality agreement	Confidentiality agreement	8 tablets/day (4*150 mg/dose, twice daily)	mPFS: 41.6 months (ALESIA clinical trial)	Government/hospital published prices, Yaozhi website (27)
Lorlatinib			1 tablet/day (1*100 mg/dose, once daily)	mPFS: >60 months (CROWN clinical trial) **	
Crizotinib			2 tablets/day (1*250 mg/dose, twice daily)	mPFS: 10.9 months (ALEX clinical trial)	
Brigatinib			1 tablet/day during the first week (190 mg/dose, once daily); 2 tablets/day thereafter (290 mg/dose, once daily)	mPFS: 24.0 months (ALTA-1 L clinical trial)	
Ensartinib			3 tablets/day (2,100 mg/dose + 125 mg/dose, once daily)	mPFS: 31.3 months (eXalt3 clinical trial)	
Ceritinib			3 tablets/day (3*150 mg/dose, once daily)	mPFS: 16.6 months (ASCEND-4 clinical trial)	
Other cost					
Additional treatment costs for brain metastases compared to non-brain metastases	33,621/person/year	—	—	—	Zhang et al. (14)
Chemotherapy follow-up costs	1,600/cycle	(1,500, 3,000)	—	4 follow-ups/year	Expert consultation
Targeted therapy follow-up costs	2,500/session	(2000, 3,000)	—	5 follow-ups/year	Expert consultation
Radiotherapy follow-up costs	2,877/course	(2,818, 2,923)	—	4 follow-ups/year	Yang et al. (29)
AEs cost for alectinib ^a^	15.37/person/month	—	—	—	Wu et al. (30)
AEs cost for alectinib ^b^	84.23/person/month	—	—	—	Camidge et al. (31)
AEs cost for lorlatinib	24.76/person/month	—	—	—	Solomon et al. (32)
AEs cost for brigatinib	170.35/person/month	—	—	—	Camidge et al. (33)
AEs cost for ensartinib	399.31/person/month	—	—	—	Luo et al. (34)
AEs cost for crizotinib	16.31/person/month	—	—	—	Solomon et al. (32)
AEs cost for ceritinib	1319.71/person/month	—	—	—	Luo et al. (34)
AEs cost for chemotherapy	68.63/person/month	—	—	—	Wu et al. (30)
AEs cost for radiotherapy	431.50/person/50Gy	—	—	—	Sun et al. (35)

* Surgical costs include surgery fees, diagnostic fees, examination fees, laboratory fees, etc. Follow-up costs include hospitalization fees, outpatient fees, etc. ** At present, the median PFS of lorlatinib has not yet been reached. The mPFS in this study is based on the latest report (60% of patients had not progressed at 60 months). ^a^ We use the adverse events observed in ALINA to estimate the AEs management cost of patients on locoregional recurrence. ^b^ We use other source to inform the adverse events realized by patients on metastatic treatment after recurrence, as ALINA does not collect information on this matter for patients on metastatic recurrence.

### 2.7 Average recurrence treatment costs per patient

Based on specific parameters, including treatment regimens, medication costs, surgical expenses, and follow-up fees, the average recurrent treatment costs per patient were calculated for both the intervention and control groups. For the intervention group, the average recurrent treatment costs were estimated at 729,048 RMB, comprising 636,837 RMB for locoregional recurrence and 799,070 RMB for metastatic recurrence. In comparison, the control group had average recurrent treatment costs of 675,176 RMB, including 541,430 RMB for locoregional recurrence and 758,300 RMB for metastatic recurrence. It should be noted that these figures represent the conditional mean cost among patients who experienced recurrence. This difference is attributed to distinct post-recurrence treatment regimens; patients who recur after receiving adjuvant alectinib may be switched to different, potentially more costly, second-line ALK inhibitors such as lorlatinib. However, despite this higher conditional cost for an individual who recurs, the overall population-level expenditure is substantially lower due to the marked reduction in the total number of recurrence events.

### 2.8 Scenario analyses

In the scenario analysis section, this study employed a series of methodological approaches to assess the robustness and reliability of the economic evaluation results. Specifically, several key parameters are varied to simulate different realistic conditions. These include altering the market share of alectinib - which is set at 75% in the base-case scenario - to 50 and 100%, respectively. Additionally, changes in the market share distribution among first-line treatment options for metastatic recurrence are incorporated. Furthermore, a sensitivity analysis on the price of alectinib is conducted by applying a ± 20% variation. These scenario-based adjustments aim to evaluate the consistency of the model outcomes under varying assumptions and to test the stability of the conclusions drawn from the base-case analysis.

## 3 Results

### 3.1 Base-case results

Over a 10-year study period, the control group received adjuvant chemotherapy exclusively and 75% of the intervention group were treated with alectinib while the remaining 25% receive chemotherapy, the intervention group demonstrated significant reductions in both locoregional and metastatic recurrence rates. The intervention group reduced the risk of disease recurrence by 45.82% versus the control group (36.52% for intervention group vs. 67.40% for control group). Over the 10 years, the intervention group achieved a cumulative reduction of 11,300 recurrence cases including 3,684 cases of locoregional recurrences and 7,616 cases of metastatic recurrences. These reductions showed a consistent year-on-year improvement (see Table 5).

**Table 5:** Recurrence cases and the cost of treatment in the intervention group and the control group under basic conditions.

Year	Recurrence cases (persons)			Cost of treatment (million RMB)		
	Intervention group	Control group	Difference	Intervention group	Control group	Difference
2025	209	510	−301	1.66	3.32	−1.66
2026	520	1,179	−659	4.24	7.75	−3.51
2027	863	1812	−949	6.50	12.01	−5.51
2028	1,209	2,364	−1,155	8.82	15.79	−6.98
2029	1,544	2,826	−1,282	11.07	18.99	−7.92
2030	1,668	3,003	−1,335	12.01	20.32	−8.31
2031	1763	3,134	−1,371	12.72	21.29	−8.57
2032	1824	3,220	−1,396	13.18	21.92	−8.75
2033	1866	3,283	−1,417	13.50	22.38	−8.89
2034	1899	3,333	−1,435	13.74	22.74	−9.01
Total	13,364	24,664	−11,300	97.43	166.53	−69.10

In addition, the intervention group demonstrated a significant reduction in recurrent treatment costs compared to the control group. Specifically, the total cost savings amounted to 6.910 billion RMB, including 1.445 billion RMB saved in locoregional recurrence treatment costs and 5.465 billion RMB saved in metastatic recurrence treatment costs. This represents a 41.49% overall reduction in recurrent treatment costs, with locoregional recurrence decreased by 28.23%, while metastatic recurrence costs experienced a more pronounced reduction of 47.38% (see Table 5).

### 3.2 Scenario analysis

In the base-case scenario, the intervention group assumes a 75% usage rate for alectinib. Scenario analysis shows that as the proportion of alectinib usage increases from 50 to 75% and subsequently to 100%, both the reductions in recurrence cases and treatment costs rise proportionally, reflecting a consistent trend. Across the three adoption scenarios (50%/75%/100%), both recurrence reductions and cost savings increase monotonically with uptake. The direction and magnitude of the economic advantage remain robust, with metastatic recurrence prevention contributing to the majority of savings in each scenario (see Table 6).

**Table 6:** Recurrence reductions and recurrence-related cost savings under alternative adjuvant alectinib uptake scenarios (10-year horizon).

Uptake of adjuvant alectinib	Recurrences averted (persons)			Cost savings (RMB, billion)		
	Locoregional	Metastatic	Total	Locoregional	Metastatic	Total
50%	2,456	5,077	7,533	0.963	3.644	4.607
75% (base)	3,684	7,616	11,300	1.445	5.465	6.910
100%	4,912	10,155	15,067	1.926	7.287	9.213

The baseline adjuvant uptake rate of 75% was selected to reflect anticipated real-world adoption, accounting for potential barriers in diagnostic testing, drug access, and reimbursement.

Additionally, adjustments to the first-line treatment regimen and its proportional usage for metastatic recurrence were evaluated based on market research data from pharmaceutical consulting firm. The evaluated regimens include chemotherapy (10%), alectinib (50.4%), lorlatinib (17.1%), brigatinib (4.5%), ensartinib (9.0%), ceritinib (4.5%), and crizotinib (4.5%). After modifying the proportion of first-line treatment regimens, the cost savings amounted to approximately 5.563 billion RMB. The scenario analysis confirms that these variations yield results consistent with the base-case findings.

After adjusting the price of alectinib, the results indicate that a 20% price increase leads to savings of approximately 7.901 billion RMB (+20%), while a 20% price reduction results in savings of around 5.919 billion RMB (−20%). These outcomes are largely consistent with the base-case scenario findings. However, it is noteworthy that price fluctuations exert a measurable, though moderate, influence on the total savings achievable through avoided recurrence.

## 4 Discussion

Alectinib, the first and only approved ALK+ targeted therapy for adjuvant treatment following surgery, demonstrates a favorable economic impact in this study. ALK+NSCLC is commonly diagnosed in younger patients, often with aggressive progression and poor prognostic outcomes. Early-stage ALK+ NSCLC patients benefit significantly from localized interventions such as surgical resection, which offers the potential for long-term survival.

Adjuvant treatment with alectinib further improves outcomes by reducing the risk of recurrence or death by 76% in patients with stage IB (tumor ≥4 cm) to stage IIIA ALK+ NSCLC. This improvement highlights the importance of equitable access to effective therapies for this rare subgroup. Compared to adjuvant chemotherapy alone, alectinib significantly lowers the 10-year recurrence rates and treatment costs for stage IB-IIIA NSCLC patients, providing both clinical and economic advantages.

It is noteworthy that while the model estimates a higher average treatment cost for an individual patient who recurs in the intervention group (RMB 729,048) compared to the control group (RMB 675,176), the total population-level costs are substantially lower. This apparent paradox is explained by alectinib’s profound efficacy in preventing recurrence. The higher per-patient cost is a conditional average reflecting different treatment patterns after recurrence, such as the use of subsequent ALK inhibitors for patients progressing on alectinib. However, because far fewer patients in the alectinib arm experience recurrence in the first place, the substantial reduction in the sheer volume of recurrence events leads to significant overall cost savings for the healthcare system.

Under the base-case scenario, there is a cumulative reduction of 11,300 recurrence cases over 10 years, along with a decrease of 6.910 billion RMB in cumulative recurrence treatment costs. Sensitivity analysis indicates that as the proportion of alectinib use increases, proportional reductions in both recurrence cases and treatment costs are observed. Overall, the findings from scenario analyses align closely with the baseline results, further reinforcing the robustness of the study.

Several limitations of this study should be acknowledged. First, the analysis assumes a 5-year disease-free survival period without recurrence, an assumption supported by some clinical experts to validate the hypothesis. Second, a key limitation and a major source of uncertainty in the model is the synthesis of data from different sources to estimate transition probabilities. Specifically, the probabilities for disease-free survival (DFS) were derived from the ALINA trial, which enrolled an adjuvant therapy population, while the progression-free survival (PFS) probabilities for post-recurrence progression were informed by the ALEX trial, which focused on a metastatic setting. This approach may introduce confounding bias due to inherent differences in the trials’ patient populations, study designs, and endpoints. Ideally, post-recurrence outcomes would be modeled using long-term follow-up data from the adjuvant ALINA cohort. However, given the immaturity of these data, synthesizing evidence from separate but clinically relevant trials is a common and necessary practice in health economic modeling. In the absence of individual patient-level data (IPD) from both trials to perform advanced adjustments such as a Matching-Adjusted Indirect Comparison (MAIC), this assumption remains a significant limitation of the model. A third limitation is that death was not modeled as an absorbing state in our model structure. This was a deliberate decision to focus the analysis strictly on the economic impact of preventing recurrence events and their associated treatments. By focusing on recurrence-related costs, we adopted a conservative approach. We acknowledge that including mortality and its associated costs would almost certainly increase the total cost savings attributed to alectinib, thereby strengthening our study’s conclusions. Future research could build upon our model to incorporate these downstream costs to provide a more comprehensive picture of the lifetime economic benefits. Finally, while costs associated with managing adverse events (AEs) were included in the model based on published literature, these estimates may not fully capture the complete real-world economic impact of treatment-related toxicity. Furthermore, other key parameters, including treatment patterns and certain costs, were derived from expert consultations, which, despite efforts to ensure representativeness, may be subject to individual biases. Nevertheless, the consultation process involved clinical experts in lung cancer from across China, helping to mitigate regional differences and enhance the generalizability of the findings.

The feasibility of implementing adjuvant alectinib as a standard of care in China is critically enhanced by its inclusion in the national reimbursement system. Following its initial inclusion for advanced NSCLC in 2019, the National Healthcare Security Administration announced on November 28, 2024, that the postoperative adjuvant therapy indication was also successfully included in the new National Basic Medical Insurance Drug Catalogue, effective January 1, 2025. This milestone establishes a clear and nationwide reimbursement pathway, significantly improving affordability and access for eligible patients. From a health system perspective, this policy is pivotal. By enabling widespread, reimbursed use of adjuvant alectinib, it facilitates the upfront investment needed to reduce long-term recurrence risk. This, in turn, is projected to decrease the downstream demand for more expensive second-line and subsequent treatments, leading to substantial net savings in overall healthcare expenditures as demonstrated in our model. Implementation feasibility is further supported by China’s expanding infrastructure for ALK molecular testing and the concentration of thoracic oncology expertise in tertiary hospitals. Nevertheless, achieving equitable access remains a challenge. Potential disparities can arise across different regions due to variations in diagnostic capabilities, provincial budget allocations for high-cost drugs, and individual hospital formulary management practices. Therefore, while the national reimbursement policy creates a realistic pathway for large-scale implementation, complementary strategies to address regional inequities - such as standardizing diagnostic services and ensuring consistent formulary access - will be crucial to maximizing the health system benefits projected by this study.

## 5 Conclusion

The use of alectinib as a postoperative adjuvant therapy significantly lowers both the recurrence rate and recurrence-related treatment costs for stage IB (tumor ≥4 cm) to IIIA ALK-positive NSCLC patients compared to platinum-based chemotherapy. From the perspective of the Chinese healthcare system, this strategy demonstrates substantial potential to reduce recurrence rates and achieve meaningful cost savings.

## Data Availability

The original contributions presented in the study are included in the article/Supplementary material, further inquiries can be directed to the corresponding authors.

## Footnotes

1 https://db.yaozh.com/.
