# Supplementary material for: The long-term avoided recurrences and recurrence-related cost of alectinib for postoperative adjuvant therapy in Chinese patients with early-stage ALK-positive non-small cell lung cancer
Source: Front Public Health. 2026 Feb 5;13:1571980. doi: 10.3389/fpubh.2025.1571980 (PMC12916615; doi:10.3389/fpubh.2025.1571980)
Supplement: Supplementary file 1 [file Data_Sheet_1.pdf]

Supplementary Material

Figure 1A. Kaplan-Meier curve of Investigator-Assessed DFS (ALINA; clinical cut-off: 26.06. 2023) 1

Figure 2A. Log-cumulative Hazard Plot – Investigator-Assessed DFS (ALINA; clinical cut-off: 26.06.2023) 1

Figure 3A. Schoenfeld Residuals – Investigator-Assessed DFS (ALINA; clinical cut-off: 26.06.2023) 2

Table 1A. Occurrence of Grade 3-5 Treatment Emergent Adverse Events during Adjuvant Treatment (ALINA; Safety Evaluable Patients; Clinical Cut-Off: 26.06.2023) 3

Table 2A. Occurrence of Grade 3-5 Treatment Emergent Adverse Events after Recurrence 5

Table 3A. Unit Cost of Managing Adverse Events 9

Table 4A.The CHEERS 2022 checklist 13

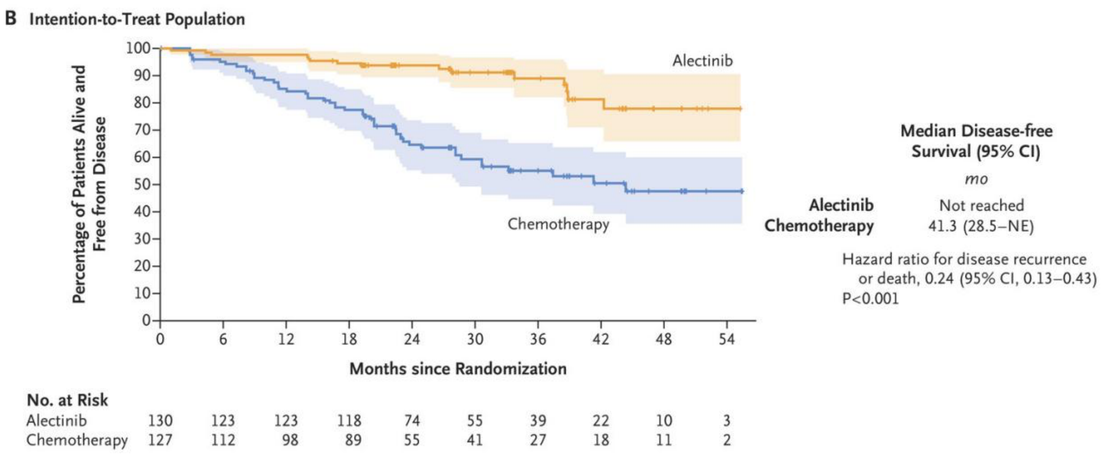

Figure 1A. Kaplan-Meier curve of Investigator-Assessed DFS (ALINA; clinical cut-off: 26.06. 2023)

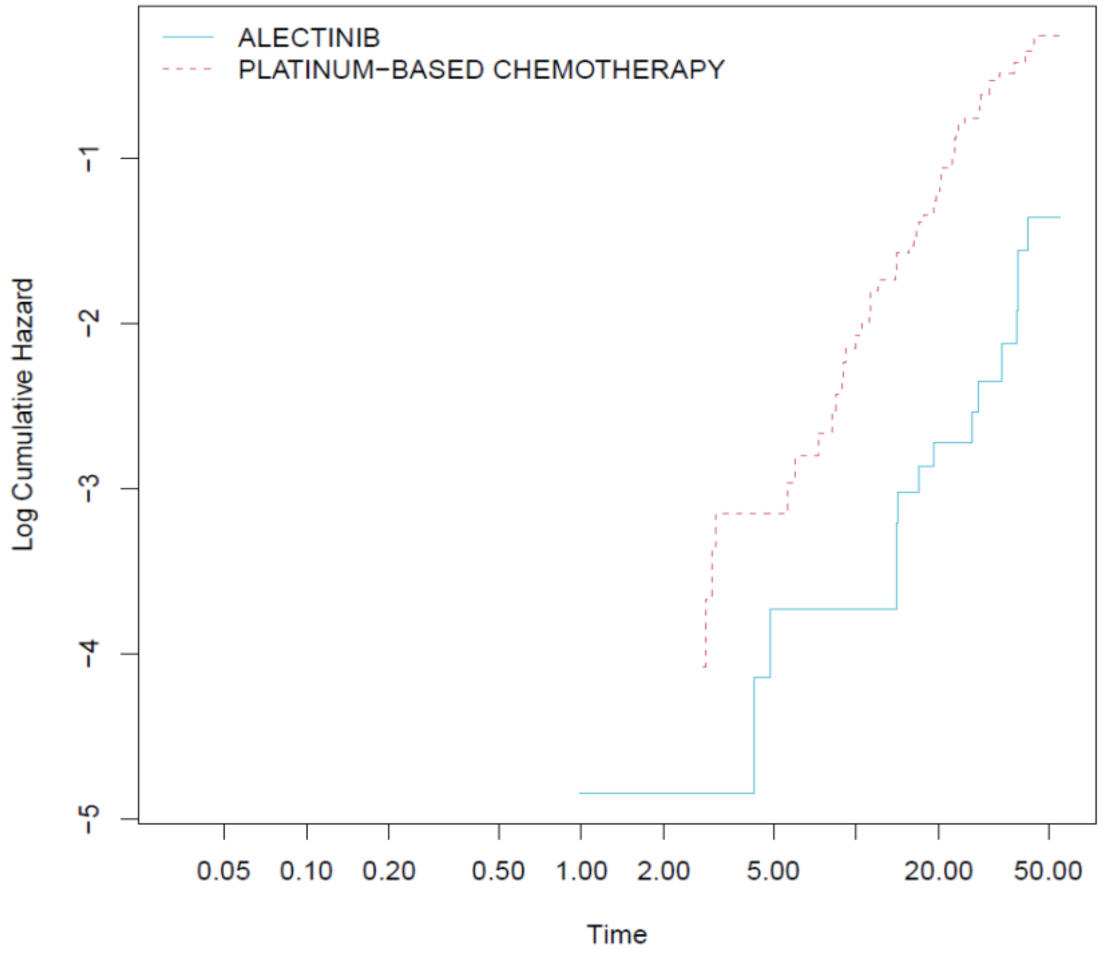

Figure 2A. Log-cumulative Hazard Plot – Investigator-Assessed DFS (ALINA; clinical cut-off: 26.06.2023)

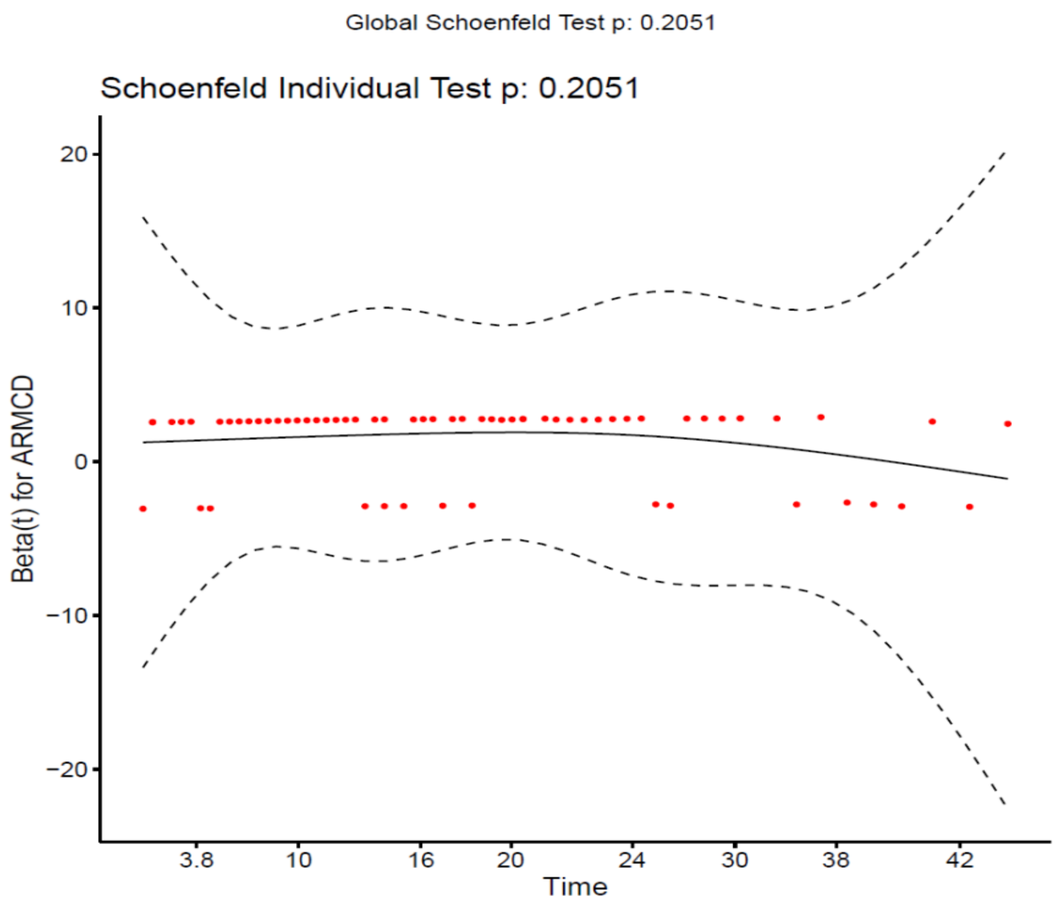

Figure 3A. Schoenfeld Residuals – Investigator-Assessed DFS (ALINA; clinical cut-off: 26.06.2023)

The ALINA does not consider grade 1-2 adverse events as these are events that are defined by mild to moderate symptoms which may not require any intervention. It only considers grade 3-5 treatment emergent adverse events as these are events that are treatment related and produce severe to life threatening symptoms that may require an invasive or emergency intervention. Table 1A present the adverse events observed in ALINA to inform their occurrence during adjuvant treatment. We use this data to calculate a monthly probability of experiencing each event while on treatment together with an estimate on total follow-up.

$$P(adverse\ event_x) = 1 - e^{-occurence_x/follow-up}$$

x is the adverse event, $occurence$ is the number of times it occurred, and $follow - up$ is follow-up in months.

Table 1A. Occurrence of Grade 3-5 Treatment Emergent Adverse Events during Adjuvant Treatment (ALINA; Safety Evaluable Patients; Clinical Cut-Off: 26.06.2023)

Adverse Events	Intervention Arm (N = 128)		Control Arm (N = 120)	
	Total Follow-Up = 4, 092 months		Total Follow-Up = 3, 842 months	
	Occurrence	Probability	Occurrence	Probability
Neutrophil count decreased	0	0.0000	12	0.0031
Blood creatine phosphokinase increased	8	0.0020	0	0.0000
White blood cell counts decreased	0	0.0000	4	0.0010
Alanine aminotransferase increased	2	0.0005	0	0.0000
Blood bilirubin increased	2	0.0005	0	0.0000
Aspartate aminotransferase increased	1	0.0002	0	0.0000
Blood creatine increased	1	0.0002	0	0.0000
Liver function test increased	1	0.0002	0	0.0000
Neutropenia	0	0.0000	10	0.0026
Anaemia	0	0.0000	1	0.0003

Febrile neutropenia	0	0.0000	1	0.0003
Leukopenia	0	0.0000	1	0.0003
Nausea	0	0.0000	5	0.0013
Constipation	1	0.0002	1	0.0003
Vomiting	0	0.0000	2	0.0005
Abdominal pain	0	0.0000	1	0.0003
Diarrhoea	1	0.0002	0	0.0000
Epigastric discomfort	0	0.0000	1	0.0003
Regurgitation	0	0.0000	1	0.0003
Stomatitis	1	0.0002	0	0.0000
Asthenia	0	0.0000	3	0.0008
Fatigue	1	0.0002	2	0.0005
Decreased appetite	0	0.0000	1	0.0003
Hypertriglyceridaemia	1	0.0002	0	0.0000
Type 2 diabetes mellitus	0	0.0000	1	0.0003
Appendicitis	1	0.0002	0	0.0000
Urinary tract infection	0	0.0000	1	0.0003
Cough	1	0.0002	0	0.0000
Pneumonitis	1	0.0002	0	0.0000
Pulmonary embolism	0	0.0000	1	0.0003
Rash	1	0.0002	0	0.0000
Rash maculo-papular	1	0.0002	0	0.0000
Embolism	0	0.0000	1	0.0003
Lymphoedema	1	0.0002	0	0.0000
Hyperbilirubinaemia	1	0.0002	0	0.0000
Myalgia	1	0.0002	0	0.0000

We use other sources to inform the adverse events realised by patients on metastatic treatment after recurrence, as ALINA does not collect information on this matter for patients on subsequent treatment after earliest contributing event. The CEA uses the same formula above to calculate a monthly probability of experiencing each of the events. A limitation with the use of these studies is that they did not present any evidence on the occurrence of grade 3-5 treatment emergent adverse events. Instead, they presented evidence on the number of patients who experienced each grade 3-5 adverse event. Additionally, the cost parameters for the management of adverse events associated with ensartinib and ceritinib were derived from Luo et al(Luo et al., 2022), while those related to radiotherapy were obtained from Sun et al (Sun et al., 2023).

Table 2A. Occurrence of Grade 3-5 Treatment Emergent Adverse Events after Recurrence

**Occurrence of Adverse Events**	**Alectinib**	**Crizotinib**	**Brigatinib**	**Lorlatinib**	**Cisplatin + Pemetrexed**
Median Follow-Up Duration	27.8 months	29.3 months	40.4 months	36.7 months	5.8 months
Sample Size	152	142	136	149	34
Definition of AEs	Number of patients with grade 3-5 adverse events reported in ≥ 2 patients	Number of patients with grade 3-4 adverse events that differed by more than 10 percentage points between treatment arms	Number of patients with treatment-emergent grade 3-5 adverse events reported in at least 2% of patients	Number of patients with grade 3-4 adverse events that differed by more than 10 percentage points between treatment arms	Number of patients with grade 3-5 adverse events occurring in more than 1 patient in either treatment arm
Alanine aminotransferase increased	7	6	6	4	n.r.

Aspartate aminotransferase increased	8	5	6	3	n.r.
Asthenia	n.r.	n.r.	n.r.	n.r.	1
Fatigue	n.r.	n.r.	n.r.	n.r.	3
Blood CPK	5	n.r.	n.r.	n.r.	n.r.
Blood bilirubin increased	3	n.r.	n.r.	n.r.	n.r.
Blood creatinine increased	2	n.r.	36	n.r.	n.r.
Gamma-glutamyltransferase increased	1	n.r.	3	n.r.	n.r.
Neutrophil count decreased	0	n.r.	1	n.r.	n.r.
Anemia	8	4	4	5	2
Neutropenia	0	n.r.	2	n.r.	4
Pulmonary embolism	2	n.r.	3	n.r.	n.r.
Pleural effusion	2	n.r.	2	n.r.	n.r.
Pneumonia	4	n.r.	7	n.r.	0
Febrile neutropenia	n.r.	n.r.	n.r.	n.r.	2
Stomatitis	n.r.	n.r.	n.r.	n.r.	2
Pneumothorax	2	n.r.	n.r.	n.r.	n.r.
Urinary tract infection	4	n.r.	1	n.r.	n.r.
Hyponatremia	3	n.r.	n.r.	n.r.	n.r.
Hypokalemia	2	n.r.	n.r.	n.r.	n.r.

Lung infection	3	n.r.	n.r.	n.r.	n.r.
Bronchitis	2	n.r.	n.r.	n.r.	n.r.
Nausea	1	3	3	1	n.r.
Vomiting	0	2	2	1	n.r.
Diarrhea	1	1	3	2	n.r.
Acute kidney injury	4	n.r.	n.r.	n.r.	0
Confusional state	1	n.r.	n.r.	n.r.	n.r.
Rash	3	n.r.	n.r.	n.r.	n.r.
Death	2	n.r.	n.r.	n.r.	n.r.
Hyperbilirubinemia	2	n.r.	n.r.	n.r.	n.r.
Arthralgia	1	0	n.r.	1	n.r.
Hypercholesterolaemia	n.r.	0	n.r.	29	n.r.
Hypertriglyceridaemia	n.r.	0	n.r.	34	n.r.
Oedema	n.r.	2	n.r.	6	n.r.
Weight increased	n.r.	3	n.r.	30	n.r.
Peripheral neuropathy	n.r.	1	n.r.	2	n.r.
Cognitive effects	n.r.	0	n.r.	5	n.r.
Hypertension	n.r.	1	19	17	n.r.
Constipation	n.r.	1	n.r.	0	n.r.
Vision disorder	n.r.	1	n.r.	0	n.r.
Mood effects	n.r.	0	n.r.	2	n.r.
Hyperlipidaemia	n.r.	0	n.r.	3	n.r.
Decreased appetite	n.r.	4	1	0	n.r.
Lipase increased	n.r.	n.r.	21	n.r.	n.r.
Amylase increased	n.r.	n.r.	8	n.r.	n.r.
Neoplasm progression	n.r.	n.r.	4	n.r.	n.r.

Blood alkaline phosphate increased	n.r.	n.r.	4	n.r.	n.r.
Dyspnea	n.r.	n.r.	3	n.r.	n.r.
Hypophosphatemia	n.r.	n.r.	3	n.r.	n.r.
Headache	n.r.	n.r.	3	n.r.	n.r.
Upper abdominal pain	n.r.	n.r.	1	n.r.	n.r.
Reference	(Camidge et al., 2019)	(Solomon et al., 2023)	(Camidge et al., 2021)	(Solomon et al., 2023)	(Novello et al., 2018)

To calculate the cost of managing adverse events, we apply the costs reported in Table 3A to the proportion of patients who experienced them.

Table 3A. Unit Cost of Managing Adverse Events

Adverse Events	Unit Cost (RMB¥)	Reference
Asthenia	869.22	(Naik et al., 2023)
Pneumonia	8,973.38	(Naik et al., 2023)
Syncope	1,000.00	(Huygens et al., 2023)
Fatigue	225.00	(Huygens et al., 2023)
Anemia	15,695.88	(Naik et al., 2023)
Neutropenia	7,988.24	(Naik et al., 2023)
Febrile neutropenia	7,988.24	(Naik et al., 2023)
Stomatitis	150.00	(Huygens et al., 2023)
Acute kidney injury	15,695.88	(Huygens et al., 2023)
Diarrhea	2,982.00	(Naik et al., 2023)
Nausea	500.00	(Huygens et al., 2023)
Vomiting	474.12	(Naik et al., 2023)
Alanine aminotransferase increased	2,135.91	(Naik et al., 2023)
Decreased appetite	100.00	(Huygens et al., 2023)
Aspartate aminotransferase increased	2,135.97	(Naik et al., 2023)
Weight decreased	20.00	(Huygens et al., 2023)
Blood alkaline phosphate increased	2,643.84	(Naik et al., 2023)
Abdominal pain	400.00	(Huygens et al., 2023)
Back pain	400.00	(Huygens et al., 2023)
Headache	400.00	(Huygens et al., 2023)

Glutamyltransferase increased	2,643.84	(Naik et al., 2023)
Pyrexia	225.00	(Huygens et al., 2023)
Abdominal pain upper	400.00	(Huygens et al., 2023)
Dyspnoea	65,332.15	(Naik et al., 2023)
Non-cardiac chest pain	400.00	(Huygens et al., 2023)
Electrocardiogram Qt prolonged	1,000.00	(Huygens et al., 2023)
Hypokalaemia	100.00	(Huygens et al., 2023)
Pain	400.00	(Huygens et al., 2023)
Pericardial effusion	1,250.00	(Huygens et al., 2023)
Pleural effusion	1,250.00	(Huygens et al., 2023)
Respiratory tract infection	600.00	(Huygens et al., 2023)
Blood lactate dehydrogenase increased	500.00	(Huygens et al., 2023)
Dehydration	250.00	(Huygens et al., 2023)
Muscular weakness	869.22	(Naik et al., 2023)
Hyperglycaemia	250.00	(Huygens et al., 2023)
Amylase increased	2,643.84	(Huygens et al., 2023)
Malaise	250.00	(Huygens et al., 2023)
Pain in extremity	400.00	(Huygens et al., 2023)
General physical health deterioration	1,000.00	(Huygens et al., 2023)
Hyponatraemia	100.00	(Huygens et al., 2023)
Chest pain	400.00	(Huygens et al., 2023)
Creatinine renal clearance decreased	400.00	(Huygens et al., 2023)
Depression	400.00	(Huygens et al., 2023)

Dysphagia	400.00	(Huygens et al., 2023)
Pericarditis	1,000.00	(Huygens et al., 2023)
Respiratory failure	65,332.15	(Naik et al., 2023)
C-reactive protein increased	400.00	(Huygens et al., 2023)
Cognitive disorder	400.00	(Huygens et al., 2023)
Epilepsy	400.00	(Huygens et al., 2023)
Lower respiratory tract infection	600.00	(Huygens et al., 2023)
Lymphocyte count decreased	1,000.00	(Huygens et al., 2023)
Myocardial ischemia	1,250.00	(Huygens et al., 2023)
Transaminases increased	2,135.91	(Naik et al., 2023)
Angina pectoris	1,000.00	(Huygens et al., 2023)
Aphasia	500.00	(Huygens et al., 2023)
Atrial flutter	1,250.00	(Huygens et al., 2023)
Biliary tract infection	600.00	(Huygens et al., 2023)
Cerebrovascular accident	15,695.88	(Naik et al., 2023)
Chronic kidney disease	1,250.00	(Huygens et al., 2023)
Deep vein thrombosis	1,250.00	(Huygens et al., 2023)
Depressed level of consciousness	500.00	(Huygens et al., 2023)
Electrocardiogram T-wave inversion	1,000.00	(Huygens et al., 2023)
Faecaloma	1,000.00	(Huygens et al., 2023)
Gastrointestinal obstruction	1,000.00	(Huygens et al., 2023)
Gastrointestinal perforation	1,000.00	(Huygens et al., 2023)
Hepatic enzyme increased	2,643.84	(Naik et al., 2023)

Hypertensive crisis	15,695.88	(Naik et al., 2023)
Hypoxia	221.00	(Huygens et al., 2023)
Interstitial lung disease	8,973.38	(Naik et al., 2023)
Jaundice	2,643.84	(Naik et al., 2023)
Lenticular opacities	2,643.84	(Naik et al., 2023)
Loss of consciousness	15,695.88	(Naik et al., 2023)
Lung infiltration	15,695.88	(Naik et al., 2023)
Mobility decreased	15,695.88	(Naik et al., 2023)
Neutrophil count decreased	7,988.24	(Naik et al., 2023)
Pathological fracture	10,000.00	(Huygens et al., 2023)
Petit mal epilepsy	500.00	(Huygens et al., 2023)
Pneumothorax	1,250.00	(Huygens et al., 2023)
Respiratory distress	65,332.15	(Naik et al., 2023)
Tumour flare	3,000.00	(Huygens et al., 2023)
Typhoid fever	600.00	(Huygens et al., 2023)
Urinary bladder rapture	2,982.00	(Naik et al., 2023)

Table 4A.The CHEERS 2022 checklist

	Item	Guidance for Reporting	Reported in section
**TITLE**			
Title	1	Identify the study as an economic evaluation and specify the interventions being compared.	Title
**ABSTRACT**			
Abstract	2	Provide a structured summary that highlights context, key methods, results and alternative analyses.	Abstract
**INTRODUCTION**			
Background and objectives	3	Give the context for the study, the study question and its practical relevance for decision making in policy or practice.	1 Induction
**METHODS**			
Health economic analysis plan	4	Indicate whether a health economic analysis plan was developed and where available.	Not performed
Study population	5	Describe characteristics of the study population (such as age range, demographics, socioeconomic, or clinical characteristics).	2.1 Target Population
Setting and location	6	Provide relevant contextual information that may influence findings.	2.2 Model Overview
Comparators	7	Describe the interventions or strategies being compared and why chosen.	2.2 Model Overview
Perspective	8	State the perspective(s) adopted by the study and why chosen.	2.2 Model Overview
Time horizon	9	State the time horizon for the study and why appropriate.	2.2 Model Overview
Discount rate	10	Report the discount rate(s) and reason chosen.	Not performed
Selection of outcomes	11	Describe what outcomes were used as the measure(s) of benefit(s) and harm(s).	2.2 Model Overview
Measurement of outcomes	12	Describe how outcomes used to capture benefit(s) and harm(s) were measured.	2.3 Calculation of Patients in Different Health States
Valuation of outcomes	13	Describe the population and methods used to measure and value outcomes.	2.4-2.7
Measurement and valuation of resources and costs	14	Describe how costs were valued.	2.4-2.7
Currency, price date, and conversion	15	Report the dates of the estimated resource quantities and unit costs, plus the currency and year of conversion.	2.6 Specific Cost Parameter Settings
Rationale and description of model	16	If modelling is used, describe in detail and why used. Report if the model is publicly available and where it can be accessed.	2.2 Model Overview, Figure 1
Analytics and assumptions	17	Describe any methods for analysing or statistically transforming data, any extrapolation methods, and approaches for validating any model used.	2.2 Model Overview, Figure 1
Characterizing heterogeneity	18	Describe any methods used for estimating how the results of the study vary for sub-groups.	Not applicable
Characterizing distributional effects	19	Describe how impacts are distributed across different individuals or adjustments made to reflect priority populations.	Not Applicable
Characterizing uncertainty	20	Describe methods to characterize any sources of uncertainty in the analysis.	2.8 Scenario Analyses
Approach to engagement with patients and others affected by the study	21	Describe any approaches to engage patients or service recipients, the general public, communities, or stakeholders (e.g., clinicians or payers) in the design of the study.	2.3-2.6
**RESULTS**			
Study parameters	22	Report all analytic inputs (e.g., values, ranges, references) including uncertainty or distributional assumptions.	Table 1, Table 2, Table 3, Table 4, and Supplementary Tables
Summary of main results	23	Report the mean values for the main categories of costs and outcomes of interest and summarise them in the most appropriate overall measure.	Table 5
Effect of uncertainty	24	Describe how uncertainty about analytic judgments, inputs, or projections affect findings. Report the effect of choice of discount rate and time horizon, if applicable.	Table 6
Effect of engagement with patients and others affected by the study	25	Report on any difference patient/service recipient, general public, community, or stakeholder involvement made to the approach or findings of the study	Not performed
**DISCUSSION**			
Study findings, limitations, generalizability, and current knowledge	26	Report key findings, limitations, ethical or equity considerations not captured, and how these could impact patients, policy, or practice.	4.Discussion
**OTHER RELEVANT INFORMATION**			
Source of funding	27	Describe how the study was funded and any role of the funder in the identification, design, conduct, and reporting of the analysis	Funding
Conflicts of interest	28	Report authors conflicts of interest according to journal or International Committee of Medical Journal Editors requirements.	Conflict of interest Section

Note: For consistency, the CHEERS statement checklist format is based on the format of the CONSORT statement checklist(Husereau et al., 2022).

References

Camidge, D.R., Dziadziuszko, R., Peters, S., Mok, T., Noe, J., Nowicka, M., et al. (2019). Updated Efficacy and Safety Data and Impact of the EML4-ALK Fusion Variant on the Efficacy of Alectinib in Untreated ALK-Positive Advanced Non-Small Cell Lung Cancer in the Global Phase III ALEX Study. *J Thorac Oncol* 14(7)**,** 1233-1243. doi: 10.1016/j.jtho.2019.03.007.

Camidge, D.R., Kim, H.R., Ahn, M.J., Yang, J.C.H., Han, J.Y., Hochmair, M.J., et al. (2021). Brigatinib Versus Crizotinib in ALK Inhibitor-Naive Advanced ALK-Positive NSCLC: Final Results of Phase 3 ALTA-1L Trial. *J Thorac Oncol* 16(12)**,** 2091-2108. doi: 10.1016/j.jtho.2021.07.035.

Husereau, D., Drummond, M., Augustovski, F., de Bekker-Grob, E., Briggs, A.H., Carswell, C., et al. (2022). Consolidated Health Economic Evaluation Reporting Standards 2022 (CHEERS 2022) Statement: Updated Reporting Guidance for Health Economic Evaluations. *Value Health* 25(1)**,** 3-9. doi: 10.1016/j.jval.2021.11.1351.

Huygens, S., Vellekoop, H., Versteegh, M., Santi, I., Szilberhorn, L., Zelei, T., et al. (2023). Cost-Effectiveness Analysis of Treating Patients With NTRK-Positive Cancer With the Histology-Independent Therapy Entrectinib. *Value Health* 26(2)**,** 193-203. doi: 10.1016/j.jval.2022.08.006.

Luo, X., Zhou, Z., Zeng, X., Peng, L., and Liu, Q. (2022). Cost-effectiveness of ensartinib, crizotinib, ceritinib, alectinib, brigatinib and lorlatinib in patients with anaplastic lymphoma kinase-positive non-small cell lung cancer in China. *Front Public Health* 10**,** 985834. doi: 10.3389/fpubh.2022.985834.

Naik, J., Beavers, N., Nilsson, F.O.L., Iadeluca, L., and Lowry, C. (2023). Cost-Effectiveness of Lorlatinib in First-Line Treatment of Adult Patients with Anaplastic Lymphoma Kinase (ALK)-Positive Non-Small-Cell Lung Cancer in Sweden. *Appl Health Econ Health Policy* 21(4)**,** 661-672. doi: 10.1007/s40258-023-00807-7.

Novello, S., Mazières, J., Oh, I.J., de Castro, J., Migliorino, M.R., Helland, Å., et al. (2018). Alectinib versus chemotherapy in crizotinib-pretreated anaplastic lymphoma kinase (ALK)-positive non-small-cell lung cancer: results from the phase III ALUR study. *Ann Oncol* 29(6)**,** 1409-1416. doi: 10.1093/annonc/mdy121.

Solomon, B.J., Bauer, T.M., Mok, T.S.K., Liu, G., Mazieres, J., de Marinis, F., et al. (2023). Efficacy and safety of first-line lorlatinib versus crizotinib in patients with advanced, ALK-positive non-small-cell lung cancer: updated analysis of data from the phase 3, randomised, open-label CROWN study. *Lancet Respir Med* 11(4)**,** 354-366. doi: 10.1016/s2213-2600(22)00437-4.

Sun, H., Wang, H., Wei, Y., Wang, H., Jin, C., and Chen, Y. (2023). Cost-effectiveness of stereotactic body radiotherapy versus conventional fractionated radiotherapy for medically inoperable, early-stage non-small cell lung cancer. *Cost Eff Resour Alloc* 21(1)**,** 46. doi: 10.1186/s12962-023-00452-w.
